# COVID-19 mimics endemic tropical diseases at an early stage: a report of two symptomatic COVID-19 patients treated in a polymerase chain reaction void zone in Cameroon

**DOI:** 10.11604/pamj.2020.37.212.25545

**Published:** 2020-11-03

**Authors:** Franklin Mogo Kom, Martin Paul Baane, Marius Mbody, Moussa Abame Sanda, Bi Ndongo Bilong, Francis Ateba Ndongo, Jean-Marc Mben II

**Affiliations:** 1 Department of Epidemiology, School of Health Science, Catholic University for Central Africa, Yaoundé, Cameroon,; 2 Clinical Research Education, Networking and Consultancy, Douala, Cameroon,; 3 Institute of Health Science, Adventist University Cosendai, Nanga-Eboko, Cameroon,; 4 Medico-Social Centre of the National Social Insurance Fund, Maroua, Cameroon,; 5 Faculty of Sciences, University of Maroua, Maroua, Cameroon,; 6 Center for Mother and Child, Chantal Biya Foundation, Yaoundé, Cameroon

**Keywords:** Health personnel, fatigue, COVID-19 infection, endemic tropical infections, quinine salts

## Abstract

At the end of December 2019, they emerged a new coronavirus (SARS-CoV-2), triggering a pandemic of an acute respiratory syndrome (COVID-19) in humans. We report the relevant features of the first two confirmed cases of COVID-19 recorded from the 29^th^ April 2020 in the Far North Region of Cameroon. We did a review of the files of these two patients who were admitted to the internal medicine ward of a medical Centre in Maroua Town, Far North Region. We present 2 cases of symptomatic COVID-19 patients, both males and health personnel, with an average age of 53 years, with no recent history of travel to a COVID-19 zone at risk and working in a then COVID-19 free region. They presented with extreme fatigue as their main symptom. Both were treated initially for severe malaria with quinine sulfate infusion with initial relief of symptoms. In the first confirmed case, at his re-hospitalization with an acute respiratory syndrome, a polymerase chain reaction (PCR) test in search of SARS-CoV-2 was requested with his results available 7 days into admission. For the second case, he had his results 48 hours on admission while he was prepared to be discharged. Both control PCR tests for COVID-19 came back negative 14 days after hospitalization. Health personnel remains a group at risk for the COVID-19 infection. The clinical manifestation at an early stage may be atypical mimicking endemic tropical infections. Also, the therapeutic potential of quinine salts in the relief of symptoms of COVID-19 is questionable and remains a subject to explore in our context.

## Introduction

Coronaviruses are a group of enveloped viruses with non-segmented, single-stranded, and positive-sense RNA genomes [1]. Many kinds of mammals, such as hedgehog, pangolin, civet, bat, and so on, can serve as storage hosts of coronavirus [2-6]. There are six coronaviruses known to infect human hosts and cause respiratory diseases [1]. Among them, severe acute respiratory syndrome coronavirus (SARS-CoV) and Middle East respiratory syndrome coronavirus (MERS-CoV) are zoonotic and highly pathogenic coronaviruses that have resulted in regional and global outbreaks [1]. Coronavirus disease (COVID-19) is an infectious disease caused by a newly discovered coronavirus called SARS-CoV-2. Noteworthy, an outbreak caused by coronavirus disease 2019 (COVID-19) occurred in Wuhan City, China, in December 2019. Subsequently, a global pandemic due to the said disease ensued. Until the end of April in Cameroon, all but one region (Far North) had recorded confirmed cases of COVID-19.

## Methods

We did a review of the files of these two patients who were admitted to the internal medicine ward of a medical Centre in Maroua Town, Far North Region. We obtained administrative authorization from the director of this hospital to get access to files and informed consent was obtained from the patients to explore their medical records.

## Results

**Case 1:** 48-year-old male health personnel who was working in a then COVID-19 free region with no history of travel to a COVID-19 high-risk territory and no known contact with a confirmed case of COVID-19 developed fatigue on the 15^th^ of April 2020. He experienced severe dizziness on the 18^th^ of April 2020 while returning home from work. He has been using barrier methods such as surgical masks and examination gloves during his medical consultations, and regularly practiced hygiene measures such as hand washing and use of alcohol-based hand sanitizer. Despite 24 hours of bed rest, the persistence of fatigue prompted him to consult on 19^th^ April 2020. He admitted having myalgia, headache, nonproductive cough and, joint ache but denied having fever, chest pain, difficulty in breathing, dysuria, frequency, vomiting, diarrhea, constipation, catarrh, neither loss of smell nor taste. The patient stays alone and denied any exposure to wild animals or visit wet markets. He does not have cardiovascular disease or any other chronic disease. On examination, he was ill-looking, in an altered general state with severe asthenia, not pale and not icteric. He took a dose of an oral anti-malarial (Athemether + Lumefantrine 80/480mg) a day prior consultation. Vital signs revealed a body temperature, 37.8°C, respiratory rate of 16 breaths per min, a pulse of 64, and BP 130/69mmHg. Chest examination revealed vesicular breath sounds with no added sounds. His 1^st^ and 2^nd^ heart sounds were heard and were normal. We made a presumptive diagnosis of Severe Malaria and the following laboratory investigations were done at admission (Table 1). He was treated thus (Table 2).

**Table 1:** laboratory data of both patients

Variable	Reference range	On admission	On readmission
**Patient 1**			
**Blood**			
Hematocrit (%)	35-55	44	43
Hemoglobin (g/dl)	12-16	13,3	13,5
White cell count (per μl)	4000-10,000	9100	10700
Differential count			
Granulocytes	1,2-6,8	1,89	9,4
Lymphocytes	1,2-3,2	1,3	0,88
Platelets	150,000-400,000	342,000	249,000
Malaria parasitemia (number/mm^3^)	0	Negative	Negative
Glucose (g/d)	0,70-0,99		0,75
Creactive protein	<6mg/dl	<6mg/dl	48mg/l
**Other test**			
Chest X ray	Normal	Normal	Bilateral interstitial infiltrates
ECG	Normal	^###^	Normal
Sputum analysis + Acid Fast Bacilli (AFB)	Negative for AFB	^###^	Negative for AFB
**Patient 2**			
**Blood**			
Hematocrit (%)	35-55	43	^**^
Hemoglobin (g/dl)	122-16	12,9	^**^
White cell count (per μl)	4000-10,000	4900	^**^
Differential count			
Granulocytes	1.2-6.8	3.65	^**^
Lymphocytes	1.2-3.2	1.05	^**^
Platelets	150,000-400,000	186000	^**^
Malaria parasitemia	0	0	^**^
Glucose (g/d)	0,70-0,99	1,26	^**^
Creactive protein	<6mg/dl	24	^**^
**Other test**			
Chest X ray	Normal	Normal	^**^
ECG	Normal	Normal	^**^

### Not done ^**^ Not applicable

**Table 2:** treatment of patients on admission and readmission

Drug	Dose	Route	Flow rate (if infusion)
**Case 1**			
**On admission**			
Quinine injectable in G5% 500cc	500mg/8hrly	IV	42 drops/minute
**On readmission**			
Quinine injectable in G5% 500cc	500mg/8hrly	IV	42 drops/minute
Amoxcillin-clavulanic Acid Injectable	1g /8hrly	IV	
Azithromycin tablets	500mg /24hrly	PO	
Omeprezole injectable	40mg /24hrly	IV	
Betamethasone injectable	4mg/12hrly	IV	
**Case 2**			
**On admission**			
Quinine injectable in G5% 500cc	500mg/8hrly	IV	42 drops/minute

On 21^st^ April 2020 (day 2 of admission) he had clinical progress marked by a significant decrease in asthenia, no respiratory symptoms, and he was discharged to continue an oral anti-malaria (Arterolane 150mg + Piperaquine 750mg 1 tablet a day for 3 days). On 22^nd^ April 2020 (about 20 hours after discharge), the patient was rushed to the emergency unit with an acute onset of respiratory distress, fever, nonproductive cough with high intensity, chest pain, and severe asthenia. Due to a suspicion of COVID-19, we immediately hospitalized him in the isolation unit with all measures of protection adopted when interacting with the patient. On general assessment, he was ill-looking, markedly confused with a Glasgow coma score of 13/15 (E4, V3, M6). Vital signs, body temperature 36.7°C, blood pressure 118/73mmHg, pulse 120 beats/minute, respiratory rate of 20 breaths/min, and oxygen saturation 94%. Chest examination revealed, decreased chest movements over both lung fields, increased tactile and vocal fremitus, and diffuse fine crackles on both lung fields. He also had tenderness on palpation of the epigastrium. A clinical diagnosis of severe pneumonia (suspected case of COVID-19). We did the following laboratory tests for the patient (Table 1). We notified the case to the competent authorities and polymerase chain reaction (PCR) for SARS-CoV-2 requested, and the patient stayed in isolation, and all protection measures ensured. Meanwhile, he was put on the following treatment (Table 2). Due to the unavailability of the PCR and virus transporting material, the patient's sample was not collected only until day 4 of admission. Following his positive clinical evolution, he was previewed for discharge but left in isolation awaiting results for PCR. On day 7 of admission, qualitative Real-time reverse transcriptase polymerase chain reaction (RT-PCR) assay was positive for SARS-CoV-2.

**Case 2:** 59-year-old male, health personnel with no history of cardiovascular disease or other chronic disease working in a then COVID-19 free zone and a history of contact with a suspected case of COVID-19 (this patient passed away 30 minutes on admission and never had his sample collected). He developed fatigue on the 27^th^ of April 2020 (7 days after contact with the suspected case). This prompted consultation on the 28^th^ April 2020, where he also complained of anorexia but denied having fever, cough, chest pain, and difficulty in breathing, dysuria, frequency, vomiting, diarrhea, constipation, catarrh, and neither loss of smell nor taste. On examination, he was ill-looking, conscious, and oriented with mild asthenia. Chest examination revealed vesicular breath sounds with no added sounds. We made the clinical diagnosis of simple malaria, and he was treated as an outpatient with an oral anti-malarial (Athemether + Lumefantrine 80/480mg) despite a negative parasitemia for malaria and bed rest. Due to persistent worsening fatigue, the patient was rushed as an emergency on the sixth day following the onset of symptoms. He also complained of generalized back pain but didn't have cough, fever, nor difficulty in breathing nor chest pain.

On examination, he was ill-looking with an altered general state by severe asthenia, Glasgow coma scale 14/15 (E 4 V 4 M6). His vital signs on admission were temperature 38.3°C, pulse 104, blood pressure 110/84mmHg, and respiratory rate of 16 breaths per minute. He had no signs of meningeal irritation. The neurological examination done was unremarkable. Musculoskeletal examination revealed a normal external back with no signs of trauma and no tenderness on palpation. The chest examination didn't reveal any abnormalities. It is worth noting that one of his colleagues, who is the patient presented above, had been diagnosed with COVID-19 during the weekend before his admission. We isolated him unit and PCR test for SARS-CoV-2 requested, and the sample collection was done. We made a clinical diagnosis of severe malaria with a differential of a suspected case of COVID-19 and patient we put him on the following treatment (Table 2). We noticed significant clinical progress marked by the resolution of fatigue and back pain on day 2 of admission, and discharge was programmed for the following day. His results for SARS-CoV-2 came back on this same day (on day 2 of admission), and he was tested positive.

## Discussion

**Diagnostic approach:** we had a diagnostic dilemma due to the atypical presentation of the patients in a zone, which was then COVID-19 free. These patients came in with extreme fatigue as their main initial presenting symptoms. Both patients had indeed been on oral anti-malaria and antipyretics, which would have probably reduced symptoms like fever. We considered severe malaria as the clinical diagnosis on initial admission in both cases as both clinically, and para-clinically, we did not find any other focus of infection in these patients. Burn out was also considered as a differential or an associated diagnosis. These patients had a very tedious month with a high patient influx and working in extremely high temperatures in the Far North at about 45-50o. Burn out could probably be associated may have created some sort of immunodeficiency to favor the onset of malaria even at low parasitemia thus their probable negative parasitemia at the onset of disease [7]. Their use of an oral anti-malarial before their admission could also explain their negative parasitemia for malaria. Furthermore, clinical improvement following treatment with parenteral antimalarial during their admissions made the diagnosis of severe malaria even more scintillating. However, for the 1^st^ case, the drastic change of his presenting symptoms at readmission coupled with para clinical findings like mild leukocytosis with lymphopenia and a significant chest X-ray made us have a high index of suspicion COVID-19.

These patients were working in a then COVID-19 free zone, however, being a health worker has been isolated as a risk factor for developing this disease [8]. Despite the urgent notification of the case and request for a PCR test in search of SARS-CoV-2 for the 1^st^ case, his sample was eventually collected 4 days after notification and results available 5 days after sample collection. Case 2 had had his colleague tested positive for SARS-CoV-2 and benefited from rapid testing as it was in line with contact tracing and testing. These are the realities we face working in resource-limited zones where even four months following the outbreak of the epidemic and about two months following the first confirmed case in Cameroon, testing of suspected cases was still very problematic. When samples are collected, however, for a test which theoretically has results available in four hours we had our results more than 48 hours after sample collection. The peculiarity of where these patients live and work is that the closest testing center for COVID-19 was found more than 250km away and virus transporting media was very limited in supply. Also, some studies have shown that a chest computerized tomography (CT) scan has a higher sensitivity than PCR in the diagnosis of COVID-19 [9, 10]. As such, we would have loved to use this tool here due to the extensive diagnostic delay of PCR results; however, this practice has been condemned by the national health authorities in Cameroon [11] and PCR remains the only diagnostic tool for this disease.

**Therapeutic approach:** the management of COVID-19 worldwide remains a challenge because no known cure and no vaccines are available. Treatment options range from simple quarantine to more invasive procedures like intubation and respiratory assistance. The national protocol adopted by the ministry of health in Cameroon proposed hydroxychloroquine and azithromycin as baseline management [12]. However, access to hydroxychloroquine became very difficult as they had been stock-outs in all pharmacies for some time now especially as these treatment was provided free of charge and restricted only for confirmed cases. The scientific committee of the hospital adopted in their monthly meeting of March that taking into consideration the health emergency we face, parenteral quinine sulfate would be used as first-line in the treatment of severe malaria in patients without allergy to quinine during this period. Quinine sulfate, amidst its efficiency in the treatment of severe malaria, has been shown to have anti-viral effects [13]. In vitro evaluation of quinine sulfate has been conducted with other viruses, such as herpes simplex virus-1 (HSV-1) and influenza A virus (IAV). Quinine sulfate at micro molar but not toxic doses reduced the number of plaques formed by HSV-1 in vitro in Vero and HaCaT cell models, although no viricidal activity was observed [14, 15]. Quinine sulfate in vitro activity also tested against IAV using viral plaque inhibition assay, when evaluating its prophylactic activity showed different effects with an EC50 within the micro molar range, depending on the viral strains [16].

We opted using parenteral quinine sulfate which has not yet shown much resistance in the treatment of severe malaria [17, 18] as 1^st^ line in our patients. Parenteral quinine sulfate would not only treat the patients for severe malaria but could have some therapeutic and prophylactic activity against SARS-CoV-2, given its action against similar clinical manifesting viruses like IAV. Due to the known antiviral activity of quinine sulfate, the scarcity of hydroxychloroquine, the complexity in clinically differentiating COVID-19 and other tropical diseases, delay in getting patients tested and eventually getting results and that, symptomatic patients with COVID-19 could present with some form of the severe disease where administration of oral medication is problematic, all suspected cases of symptomatic COVID-19 in this center would be treated with parenteral quinine infusion and azithromycin at baseline while waiting for either sample collection or results understanding the considerable delay. Quinine sulfate is a low cost readily available anti-microbial agent, with a good therapeutic observance in Cameroon. Furthermore, due to the temporary resolution of the patients during their admission while singly on parenteral quinine infusion, we got even more convinced that quinine sulfate could have some effects on the SARS-CoV-2.

**Contact tracing:** for case 1, testing of colleagues in the same health institution where he works, 4/15 health personnel tested were positive for SARS-CoV-2. However, testing of 15 members of his family all came back negative. Case two was a contact of case one, and screening of 2 family members all came back negative.

## Conclusion

In this case series, we report clinical and therapeutic data on the first two cases of COVID-19 recorded in Maroua, Cameroon. We recognize the fact that we provided results based on a small number of cases, however, the comprehensive follow up of the patients enabled us to illustrate the different courses of the disease we observed, and we provided some relevant data regarding the management of the disease. These findings will contribute to a better understanding of the natural history of the disease and help in advances in the management of the disease.

## What is known about this topic

- Testing of suspected cases of COVID-19 using the Gold standard RT-PCR is still problematic in Africa;
- Health personnel remain a high-risk group for COVID-19.

### What this study adds

- The main interest of this case series is the atypical presentation of COVID-19 in these patients, with extreme fatigue being the primary symptom;
- The therapeutic potential of quinine salts for relief of symptoms in patients with COVID-19 is questionable and remains a subject to explore in our context.
